# AI’s Accuracy in Extracting Learning Experiences From Clinical Practice Logs: Observational Study

**DOI:** 10.2196/68697

**Published:** 2025-10-15

**Authors:** Takeshi Kondo, Hiroshi Nishigori

**Affiliations:** 1 Center for Medical Education, Nagoya University Graduate School of Medicine, 65, Tsurumai-cho, Showa-ku, Nagoya city, Aichi, 466-8560, Japan, +81 052 7412111; 2 The School of Health Professions Education, Maastricht University, Maastricht, The Netherlands

**Keywords:** large language models, ChatGPT, workplace-based assessment, artificial intelligence, AI

## Abstract

**Background:**

Improving the quality of education in clinical settings requires an understanding of learners’ experiences and learning processes. However, this is a significant burden on learners and educators. If learners’ learning records could be automatically analyzed and their experiences could be visualized, this would enable real-time tracking of their progress. Large language models (LLMs) may be useful for this purpose, although their accuracy has not been sufficiently studied.

**Objective:**

This study aimed to explore the accuracy of predicting the actual clinical experiences of medical students from their learning log data during clinical clerkship using LLMs.

**Methods:**

This study was conducted at the Nagoya University School of Medicine. Learning log data from medical students participating in a clinical clerkship from April 22, 2024, to May 24, 2024, were used. The Model Core Curriculum for Medical Education was used as a template to extract experiences. OpenAI’s ChatGPT was selected for this task after a comparison with other LLMs. Prompts were created using the learning log data and provided to ChatGPT to extract experiences, which were then listed. A web application using GPT-4-turbo was developed to automate this process. The accuracy of the extracted experiences was evaluated by comparing them with the corrected lists provided by the students.

**Results:**

A total of 20 sixth-year medical students participated in this study, resulting in 40 datasets. The overall Jaccard index was 0.59 (95% CI 0.46-0.71), and the Cohen κ was 0.65 (95% CI 0.53-0.76). Overall sensitivity was 62.39% (95% CI 49.96%-74.81%), and specificity was 99.34% (95% CI 98.77%-99.92%). Category-specific performance varied: symptoms showed a sensitivity of 45.43% (95% CI 25.12%-65.75%) and specificity of 98.75% (95% CI 97.31%-100%), examinations showed a sensitivity of 46.76% (95% CI 25.67%-67.86%) and specificity of 98.84% (95% CI 97.81%-99.87%), and procedures achieved a sensitivity of 56.36% (95% CI 37.64%-75.08%) and specificity of 98.92% (95% CI 96.67%-100%). The results suggest that GPT-4-turbo accurately identified many of the actual experiences but missed some because of insufficient detail or a lack of student records.

**Conclusions:**

This study demonstrated that LLMs such as GPT-4-turbo can predict clinical experiences from learning logs with high specificity but moderate sensitivity. Future improvements in AI models, providing feedback to medical students’ learning logs and combining them with other data sources such as electronic medical records, may enhance the accuracy. Using artificial intelligence to analyze learning logs for assessment could reduce the burden on learners and educators while improving the quality of educational assessments in medical education.

## Introduction

### Background

To improve the quality of education in clinical settings, it is important to understand what learners experience and how they learn [1,2]. Various workplace-based assessment tools have been developed and used to enable educators to track learners’ progress and provide feedback [3]. However, the rigorous management of learners’ progress requires frequent observation of learners, frequent evaluations, and feedback from educators. This can impose a high burden on both learners and educators, potentially hindering learning [4,5]. Thus, the challenge is accurately monitoring learning in clinical settings without burdening learners or educators.

Learners in clinical settings often document their learning and practice experiences. If these records can be analyzed to understand learners’ contexts, monitoring their learning without imposing additional burdens may be possible. One such record kept by learners during clinical clerkship is a logbook. The logbook documents the cases encountered, procedures performed, and learners’ reflections. It serves as a tool for prompting student reflections and facilitating feedback and dialogue between educators and learners [6-8]. Evaluating these records against curriculum competencies and goals without adding an extra burden on learners can help monitor their progress [9]. However, educators may have to manually match and analyze these records, which may be a significant burden [5].

Artificial intelligence (AI)–assisted text extraction and standard matching could be useful in this context. Previous studies have successfully used natural language processing, a branch of AI, to analyze supervisory feedback comments and predict student performance against competency standards [10]. AI models that integrate multiple information sources to represent student performance have also been developed [11]. Among AI technologies, large language models (LLMs) have gained attention in medical education because of their extensive pretraining on large datasets, allowing them to handle various situations, including multilingual support, with minimal adjustment [12]. Research using ChatGPT, an LLM, has shown that it can apply codes to interview texts using a codebook, suggesting its potential for extracting competency-based evaluations from student descriptions [13]. However, owing to a lack of such research, aggregation accuracy remains uncertain. Determining the extent to which LLMs can aggregate items related to curriculum goals from learner descriptions may open up opportunities to leverage LLMs to monitor learner progress and enhance education quality.

In Japanese undergraduate education, the Model Core Curriculum for Medical Education (MCC) [14] was established to define two-thirds of the undergraduate curriculum and is used as a guideline for undergraduate medical education. The MCC outlines the experiences that medical students should have by the time they graduate, focusing primarily on clinical clerkships [14]. In Japanese clinical clerkships, medical students are partially observed directly by supervisors [15], but it is difficult for busy supervisors to grasp the full scope of experiences that medical students encounter [14]. If experiences could be understood through analysis of learning logs kept by medical students, valuable information for improving the learning environment could be obtained.

### Objectives

Therefore, this study focused on undergraduate clinical clerkships in Japan to investigate the accuracy with which LLMs can aggregate goals from records kept for learning. Our research question was as follows: how accurately can an LLM predict experiences related to the goals defined by the MCC from the records that students keep for learning during clinical clerkships?

## Methods

### Context

This study was conducted as part of the participatory clinical clerkship at the Nagoya University School of Medicine, a program designed to provide medical students with practical experience in clinical settings. During the final year of medical school (sixth year), students participate in this program for 4 weeks, recording their daily experiences and learning activities. A trial to transform these records into an electronic portfolio began in 2024. This study was part of this trial.

### Dataset

This study used learning log data from sixth-year medical students to extract their experiences related to core curriculum goals. Learning log data consisted of daily records of experiences and learning activities entered by medical students into an electronic portfolio during a clinical clerkship from April 22, 2024, to May 24, 2024. The data were treated as weekly datasets.

### Extraction of Experiences

The template for extracting experiences from the dataset was the MCC [14]. This study used a table of symptoms, examinations, and procedures that medical students are expected to encounter in patients during their clinical clerkship at Nagoya University School of Medicine as the template for experience extraction (Textbox 1).

Textbox 1. **Symptoms, examinations, and procedures that medical students are expected to encounter in patients.**

**Symptoms**

- Fever
- General malaise
- Anorexia
- Weight loss
- Weight gain
- Altered mental status
- Syncope
- Seizure
- Vertigo and dizziness
- Edema
- Rash
- Cough and sputum production
- Blood in sputum and hemoptysis
- Dyspnea
- Chest pain
- Palpitations
- Dysphagia
- Abdominal pain
- Nausea and vomiting
- Hematemesis
- Melena
- Constipation
- Diarrhea
- Jaundice
- Abdominal distention and abdominal mass
- Lymphadenopathy
- Abnormal urine output or urination
- Hematuria
- Menstrual abnormality
- Anxiety or depression
- Cognitive dysfunction
- Headache
- Skeletal muscle paralysis or muscle weakness
- Gait disturbance
- Sensory disturbance
- Back pain
- Arthralgia or joint swelling

**Examinations**

- Full blood count
- Blood biochemistry
- Coagulation or fibrinolysis
- Immunoserology tests
- Urinalysis
- Stool (fecal) examination
- Blood typing (ABO, and RhHD), blood compatibility test (cross-matching), and atypical antibody screening
- Arterial blood gas analysis
- Pregnancy test
- Microbiological tests (bacterial smear, culture, identification, and antibiotic sensitivity test)
- Cerebrospinal fluid
- Pleural fluid analysis
- Peritoneal fluid analysis
- Histopathology and cytology (including intraoperative rapid diagnosis)
- Genetic testing and chromosome analysis
- Electrocardiography (ECG)
- Lung function tests
- Endocrine and metabolic function tests
- Electroencephalography
- Ultrasound
- X-ray
- Computed tomography
- Magnetic resonance imaging
- Nuclear medicine examination
- Endoscopy

**Procedures**

- Position change and transfer
- Skin antisepsis
- Application of topical medications
- Airway suction
- Nebulizer
- Venous blood sampling
- Peripheral venous catheterization
- Insertion and extraction of nasogastric tube
- Insertion and extraction of urinary catheter
- Intradermal injection
- Subcutaneous injection
- Intramuscular injection
- Intravenous injection
- Urinalysis (including pregnancy test)
- Microbiological testing (including Gram staining)
- Recording of a 12-lead ECG
- Rapid bedside ultrasound (including focused assessment with sonography for trauma [FAST]) for clinical decision-making
- Rapid antigen or pathogen testing
- Blood glucose test
- Aseptic technique
- Surgical hand washing
- Gowning techniques in the operating room
- Basic sutures and suture removal

OpenAI’s ChatGPT, Google’s Gemini, and Anthropic’s Claude were considered for the LLMs used in experience extraction. Trial prompts and randomly selected student records were entered into each web platform, and the extracted results were compared in terms of validity. Validity was evaluated from the perspective of whether the output followed the expected format, whether the output matched the experience items expected from the text, and whether the output was reproducible. ChatGPT by OpenAI produced the most valid outputs, so it was selected for this study.

LLMs, including ChatGPT, receive text data as input and generate subsequent text based on these data. Therefore, the prompt given to the LLM is crucial. In this study, prompts were created using medical students’ learning log data, which were provided to ChatGPT to extract their experiences from the logs. Experiences were extracted based on a table of symptoms, examinations, and procedures that students were expected to experience, with ChatGPT outputting a list of symptoms, examinations, and procedures inferred from the text data. To automate this process, a web application using GPT-4-turbo was developed, which allowed medical students to input learning log data and receive the extracted experiences as a list output from GPT-4-turbo (gpt-4-0125-preview). The prompt used for GPT-4-turbo and the web application code are provided in Multimedia Appendix 1.

### Evaluation of Extracted Experiences

The extracted experience goals were presented to the medical students via email. Students were asked to compare the list with their actual experiences, including those not recorded in their reflections, and submit a corrected list. The corrected lists were compared with the original learning log data to evaluate the accuracy of the extracted experiences.

### Data Analysis

The accuracy of the extracted experience goals was evaluated using the R software (version 4.1.2; R Foundation for Statistical Computing). The agreement rate between the extracted and corrected experience goals was calculated, and the accuracy of the extracted experience goals was assessed based on this agreement rate.

### Ethical Considerations

This study was approved by the ethics committee of Nagoya University Graduate School of Medicine (approval 2023-0451 31742). All participants were informed about the study’s purpose, methods, risks, and benefits and were allowed to opt out. All data were fully anonymized and handled to prevent the identification of individuals. No compensation was provided to participants in this study.

## Results

### Study Period, Participants, and Data Characteristics

During the clinical participation-based clerkship at Nagoya University Hospital from April 22, 2024, to May 24, 2024, a total of 61% (20/33) of the sixth-year students who made entries in the e-portfolio participated in the study, yielding 40 data points. All records were written in Japanese, with an average letter count of 446.2 (SD 353.52; range 72-1473). The predicted and actual experiences are shown in Table 1.

**Table 1.** Predicted and actual experience items.

Record index	Predicted item	Actual item	Number of matches	Number of experienced items extracted by GPT-4-turbo	Number of items that the students marked as experiences they had
1	Skeletal muscle paralysis or muscle weakness, gait disturbance, and sensory disturbance	Skeletal muscle paralysis or muscle weakness, gait disturbance, and sensory disturbance	3	3	3
2	Endocrine and metabolic function tests	Endocrine and metabolic function tests	1	1	1
3	Fever	Fever	1	1	1
4	Basic sutures and suture removal	Basic sutures and suture removal	1	1	1
5	Seizure, electroencephalography, and MRI^a^	Aseptic technique, electroencephalography, MRI, weight gain, and seizure	3	3	5
6	Skeletal muscle paralysis or muscle weakness, gait disturbance, and sensory disturbance	Skeletal muscle paralysis or muscle weakness, gait disturbance, and sensory disturbance	3	3	3
7	Anorexia, abdominal distention and abdominal mass, and ultrasound	Palpitations and skeletal muscle paralysis or muscle weakness	0	3	2
8	Venous blood sampling	Venous blood sampling	1	1	1
9	Rapid bedside ultrasound (including FAST^b^) for clinical decision-making and ultrasound	Skin antisepsis, rapid bedside ultrasound (including FAST) for clinical decision-making, aseptic technique, surgical handwashing, gowning techniques in the operating room, basic sutures and suture removal, ultrasound, fever, and diarrhea	2	2	9
10	Basic sutures and suture removal	Basic sutures and suture removal	1	1	1
11	Basic sutures and suture removal	Surgical handwashing, gowning techniques in the operating room, basic sutures and suture removal, full blood count, blood biochemistry, coagulation and fibrinolysis, histopathology and cytology (including intraoperative rapid diagnosis), x-ray, CT^c^, MRI, general malaise, and weight loss	1	1	12
12	Venous blood sampling and pregnancy test	Venous blood sampling	1	2	1
13	Surgical handwashing, gowning techniques in the operating room, and basic sutures and suture removal	Surgical handwashing, gowning techniques in the operating room, and basic sutures and suture removal	3	3	3
14	Pregnancy test and basic sutures and suture removal	Position change and transfer, insertion and extraction of a urinary catheter, surgical handwashing, gowning techniques in the operating room, basic sutures and suture removal, histopathology and cytology (including intraoperative rapid diagnosis), MRI, and abdominal distention and abdominal mass	1	2	8
15	Surgical handwashing	Surgical handwashing, gowning techniques in the operating room, basic sutures and suture removal, full blood count, blood biochemistry, histopathology and cytology (including intraoperative rapid diagnosis), ultrasound, and x-ray	1	1	8
16	Microbiological tests (bacterial smear, culture, identification, and antibiotic sensitivity test), nuclear medicine examination, general malaise, cough and sputum production, dyspnea, abdominal pain, nausea and vomiting, and abnormal urine output or urination	General malaise and edema	1	8	2
17	Surgical handwashing	Aseptic technique, surgical handwashing, gowning techniques in the operating room, basic sutures and suture removal, and cough and sputum production	1	1	5
18	Fever, urinalysis, microbiological tests (bacterial smear, culture, identification, and antibiotic sensitivity test), nausea and vomiting, and hematuria	Full blood count, blood biochemistry, immunoserology tests, urinalysis, microbiological tests (bacterial smear, culture, identification, and antibiotic sensitivity test), edema, palpitations, hematuria, and back pain	3	5	9
19	Blood glucose test and endocrine and metabolic function tests	Blood glucose test and endocrine and metabolic function tests	2	2	2
20	Cognitive dysfunction	Cognitive dysfunction	1	1	1
21	Chest pain	Chest pain	1	1	1
22	Surgical handwashing and basic sutures and suture removal	Aseptic technique, surgical handwashing, gowning techniques in the operating room, and basic sutures and suture removal	2	2	4
23	Cognitive dysfunction, abnormal urine output or urination, and urinalysis	Cognitive dysfunction and abnormal urine output or urination	2	3	2
24	Dyspnea	Dyspnea	1	1	1
25	Gowning techniques in the operating room	Position change and transfer, skin antisepsis, aseptic technique, surgical handwashing, gowning techniques in the operating room, and basic sutures and suture removal	1	1	6
26	Full blood count and blood biochemistry	Full blood count, blood biochemistry, immunoserology tests, and edema	2	2	4
27	CT, MRI, and x-ray	Position change and transfer, full blood count, arterial blood gas analysis, ultrasound, x-ray, CT, MRI, skeletal muscle paralysis or muscle weakness, gait disturbance, and back pain	3	3	10
28	Endocrine and metabolic function tests	Endocrine and metabolic function tests	1	1	1
29	Basic sutures and suture removal	Position change and transfer, surgical handwashing, gowning techniques in the operating room, and basic sutures and suture removal	1	1	4
30	Weight loss and skeletal muscle paralysis or muscle weakness	Blood glucose test, weight loss, and skeletal muscle paralysis or muscle weakness	2	2	3
31	Ultrasound and endoscopy	Ultrasound and endoscopy	2	2	2
32	Basic sutures and suture removal	Skin antisepsis, aseptic technique, surgical handwashing, gowning techniques in the operating room, basic sutures and suture removal, full blood count, blood biochemistry, coagulation or fibrinolysis, immunoserology tests, histopathology and cytology (including intraoperative rapid diagnosis), ultrasound, x-ray, and headache	1	1	13
33	Skin antisepsis and position change and transfer	Skin antisepsis and position change and transfer	2	2	2
34	Back pain	Weight loss, cognitive dysfunction, skeletal muscle paralysis or muscle weakness, sensory disturbance, and back pain	1	1	5
35	Arterial blood gas analysis, peripheral venous catheterization, insertion and extraction of a nasogastric tube, insertion and extraction of a urinary catheter, aseptic technique, surgical handwashing, gowning techniques in the operating room, and basic sutures and suture removal	Peripheral venous catheterization, aseptic technique, full blood count, blood biochemistry, coagulation or fibrinolysis, arterial blood gas analysis, pleural fluid analysis, ultrasound, x-ray, CT, and endoscopy	3	8	11
36	Weight gain, endocrine and metabolic function tests, and blood glucose test	Blood glucose test, full blood count, blood biochemistry, urinalysis, stool (fecal) examination, endocrine and metabolic function tests, ultrasound, CT, and weight gain	3	3	9
37	Endoscopy	Endoscopy	1	1	1
38	X-ray	X-ray and cough and sputum production	1	1	2
39	Abdominal pain	ID not found	0	1	1
40	Skin antisepsis	Skin antisepsis	1	1	1

a MRI: magnetic resonance imaging.

b FAST: focused assessment with sonography for trauma.

c CT: computed tomography.

The predicted items were experience items extracted using GPT-4-turbo from the students’ practice records. The actual items were those that the students marked as experiences they had during that period. The English-translated version of the students’ records used by GPT-4-turbo to extract experiences can be found in Multimedia Appendix 1, with the “Index” column in Table 1 corresponding to the “Index” column in Multimedia Appendix 1.

### Agreement Between LLM Predictions and Student-Reported Experiences

The Jaccard index was 0.59 (95% CI 0.46-0.71), indicating moderate agreement, and the Cohen κ was 0.65 (95% CI 0.53-0.76), indicating substantial agreement. Sensitivity and specificity were 62.39% (95% CI 49.96%-74.81%) and 99.34% (95% CI 98.77%-99.92%), respectively. The sensitivity and specificity of the LLM for each category were as follows: 45.43% (95% CI 25.12%-65.75%) and 98.75% (95% CI 97.31%-100%) for symptoms, 46.76% (95% CI 25.67%-67.86%) and 98.84% (95% CI 97.81%-99.87%) for examinations, and 56.36% (95% CI 37.64%-75.08%) and 98.92% (95% CI 96.67%-100%) for procedures, respectively. There was no significant variation among the categories. However, when calculating by category, the sensitivity tended to be lower than the overall calculation, likely due to the influence of items that were not extracted at all. The correlation between the number of characters in the students’ records and sensitivity and specificity was 0.04 and –0.64, respectively, indicating a negligible correlation with sensitivity and a moderate negative correlation with specificity. The correlation coefficients for the Jaccard index and the Cohen κ were 0.06 and –0.07, respectively, showing negligible correlations with record length.

### Patterns of Missed Experiences

There were several patterns in experiences that were not captured by GPT-4-turbo’s analysis even though students considered to have had those experiences. In this paragraph, we explain these patterns with examples corresponding to specific entries in Table 1. Due to the large volume of student records, the full texts are provided in Multimedia Appendix 2 rather than Table 1. One pattern was when predictable experiences were not picked up by GPT-4-turbo’s analysis. For example, a student (index 19 in Table 1) described encountering a case of hereditary amyotrophic lateral sclerosis, but GPT-4-turbo’s analysis failed to capture the student’s experience with muscle weakness, a symptom of amyotrophic lateral sclerosis. Another pattern was when insufficient description made prediction difficult. In total, 20% (8/40) of the students (indexes 9, 11, 15, 17, 22, 25, 29, and 32 in Table 1) recorded observing surgery, but it was unclear from the description whether they assisted in the surgery or merely observed, making it difficult for GPT-4-turbo to extract related procedures such as surgical handwashing and gowning techniques. A third pattern was when experiences were not recorded by the students, making prediction impossible. For instance, a student recorded observing a surgery (index 15 in Table 1) but actually performed suturing, an experience not captured by GPT-4-turbo due to lack of record. Similarly, a student (index 30 in Table 1) noted examining a patient with diabetes but did not record performing computed tomography or ultrasound examinations.

## Discussion

### Principal Findings

In this study, we analyzed the records kept by medical students during their clinical clerkship for learning purposes using GPT-4-turbo to predict the clinical procedures they experienced. The experiences extracted by GPT-4-turbo were evaluated for accuracy after being revised by the medical students. The extraction of experiences by GPT-4-turbo showed a sufficient level of agreement with the items that students actually experienced and demonstrated high specificity. The high specificity suggests that the extracted experiences likely mirror what the students actually encountered. However, the low sensitivity indicates that some experiences that students actually had were not captured by GPT-4-turbo’s analysis of the records. There were three main reasons why certain experiences could not be extracted: (1) experiences that could have been predicted by GPT-4-turbo’s analysis were not identified; (2) the descriptions were insufficient, making prediction difficult; and (3) there were experiences that students did not record at all.

### Implications of Findings

The results of this study suggest that LLMs such as GPT-4-turbo are able to extract experiences from learning records with sufficient accuracy. On the other hand, when the content of the learning records is insufficient or when students do not record their experiences, experience extraction becomes difficult, indicating that improving the accuracy of LLMs alone may not be sufficient.

### Comparison to the Literature

Comparison with previous studies suggests that LLMs are making it easier and more accurate to extract experiences from learning records. Unlike previous studies [10,11], which required extensive pretraining on large text datasets, this study was able to extract experiences from learning records using only prompt engineering without additional training. A related study using LLMs investigated how well GPT-3 could extract predefined codes from documents and compared its results to those of human coders [13]. In that study, providing 5 examples for each code resulted in a Cohen κ of 0.61 for some codes, although the Cohen κ for most codes was lower. In contrast, our study used GPT-4-turbo to extract experiences from learning records without providing specific examples, achieving a Cohen κ of 0.65. Although direct comparison is difficult due to differences in study targets, GPT-4-turbo may have achieved higher extraction accuracy. These findings indicate that, with the advent and evolution of LLMs, extracting experiences from learning records is becoming easier and potentially more accurate.

In addition, this study demonstrates that performance monitoring is possible by analyzing narrative records primarily intended for student learning using LLMs rather than aggregating list-based records mainly used for evaluation, as in some previous studies. Previous studies have explored the use of logbooks to monitor learners’ progress of learning. Attempts have been made to monitor skills and experiences using logbooks [16], track the progress of entrustable professional activities [8], and count the cases encountered [7]. However, the “logbooks” used in these studies were lists of cases experienced or evaluations rather than detailed descriptions of experiences [7,8]. This format is more useful for evaluation purposes rather than for recording learning, which ultimately adds to the burden on learners. Our study suggests that analyzing reflections purely recorded for learning purposes can also extract experiences, offering a technique that monitors learning situations while reducing the burden on learners and educators.

### Future Directions

While this study demonstrated the usefulness of experience extraction by LLMs, it also highlighted new challenges. GPT-4-turbo failed to extract some experiences that students actually had. The first pattern involved experience items that could not be extracted despite being predictable from the learning log content. The second pattern involved cases in which descriptions were ambiguous, making inference difficult. The third pattern involved experiences that medical students believed they had but were not recorded in learning logs, making inference impossible. Regarding the first pattern, insufficient reasoning ability of GPT-4-turbo is considered the cause. However, the reasoning ability of LLMs is improving with model evolution [17], and future improvements in LLM accuracy may partially address this issue. Regarding the second and third patterns, insufficient content in medical students’ learning logs appears to be the cause, resulting in inadequate information to infer students’ experiences. To address these challenges, it may be necessary to enrich students’ learning records or extract experiences from other sources.

**Figure 1.**
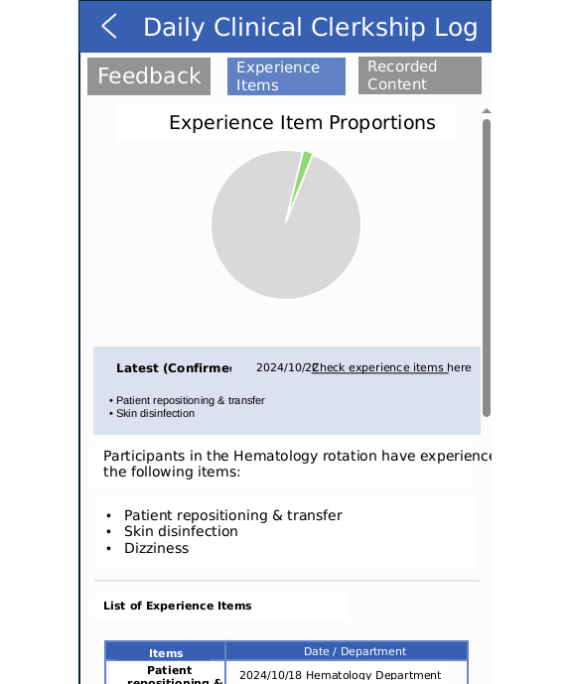
Development version of the web application.

To enhance the quality of medical students’ learning records, providing feedback using the list of experiences extracted by LLMs may be beneficial. Previous studies have shown that logbooks are useful for performance monitoring and improving educational quality, but they have also pointed out that the quality of the records is often insufficient and that feedback is needed [18]. In this study, medical students reviewed the list of experiences extracted from their learning records by GPT-4-turbo and added items that they had actually experienced but were not extracted. Since missing or incomplete records can be a reason for experiences not being extracted, this review process may serve as feedback for students, helping them reflect on what they failed to document in their records. As shown in the development version of the web application in Figure 1, displaying experience items extracted from learning logs might motivate students to improve their learning log documentation.

Combining other data such as electronic health records written by the students might be effective for more accurate monitoring of medical students’ performance. Feeding both learning logs and electronic health record descriptions into GPT-4-turbo could enhance the accuracy of experience extraction. Such an approach could lead to more accurate assessment of medical students without increasing the burden on students or faculty. However, since many LLMs, including GPT-4-turbo, are cloud-based, privacy concerns may arise [19,20]. Therefore, new approaches will need to be developed to address these privacy issues in the future.

### Limitations

This study has several limitations. First, this study used learning log data from clinical participation-based clerkships at a single university; therefore, its generalizability to learning log data from other universities or clinical clerkships is not guaranteed. In addition, the data collection period was limited to 1 month, which may not capture the full range of experiences or seasonal variations in clinical activities. While the accuracy of the extracted experience content was evaluated by using learning log data recorded by medical students and asking them to make corrections, the quality and quantity of the learning log data recorded by the students could affect the accuracy of the extracted experience content. Large-scale collaborative studies across multiple institutions and over longer periods are needed to ensure broader generalizability. Furthermore, this study used a list of symptoms, examinations, and procedures in the MCC as a template for extracting experience content; however, the results of using other templates were not examined. Future research is needed to assess performance using other evaluation criteria. Although we confirmed the correlation between record length and extraction sensitivity and specificity, we did not quantitatively evaluate the quality of the records. Future work should investigate the relationship between record quality and extraction performance. In this study, the accuracy of the extracted experience content was evaluated by using learning log data recorded by medical students and asking them to make corrections, but no strict criteria were set for what constitutes “experience” when students made corrections. Moreover, students’ judgments about whether they had actually experienced a procedure are subjective, and they may have overreported certain experiences or overlooked ones they truly had. In the clinical clerkship that served as this study’s setting, supervising physicians did not continuously monitor students, so only the students themselves could verify their experiences. Therefore, we had to rely on students’ subjective reports. In future work, it will be desirable to establish more objective evaluation criteria to reduce potential bias.

### Conclusions

In this study, records kept by medical students for learning during clinical clerkships were analyzed using GPT-4-turbo to predict experienced clinical activities. The high specificity of the GPT-4-turbo predictions suggests that the extracted experiences are likely what students actually encountered. However, the low sensitivity indicates that some actual student experiences were not captured by the GPT-4-turbo analysis. Future improvements in AI model performance, providing feedback to medical students on their records and combining learning logs with other data sources such as electronic medical records, may enhance accuracy. Analyzing records using AI may enable detailed assessments while avoiding excessive burdens on learners and educators. 

## Supplementary material

10.2196/68697 Multimedia Appendix 1 The prompts for the OpenAI application programming interface (API) and GitHub repository include an experience extraction API, prompt used in API, R code to analyze the data, and the data themselves.

10.2196/68697 Multimedia Appendix 2 Student records.

## Abbreviations

AI: artificial intelligence
LLM: large language model
MCC: Model Core Curriculum for Medical Education
