# Supplementary material for: AI’s Accuracy in Extracting Learning Experiences From Clinical Practice Logs: Observational Study
Source: JMIR Med Educ. 2025 Oct 15;11:e68697. doi: 10.2196/68697 (PMC12529426; doi:10.2196/68697)
Supplement: Multimedia Appendix 1 [file mededu-v11-e68697-s001.docx]

The following GitHub repository contains the prompts used for the OpenAI API in this study, the code for the API used in data collection, the raw data of the study results, as well as the analysis code and its outputs.

<https://github.com/ncukondo-research/extract-core-curriculum-ids-from-text>

The following is a translated prompt. For the Japanese prompt used in the API, please refer to <https://github.com/ncukondo-research/extract-core-curriculum-ids-from-text/blob/main/prompts/extract_id_ja.ts> .

| I will present a medical student's clinical training record and the objectives a medical student should experience during clinical training. Please read the clinical training record of the medical student and output the objectives the student experienced, using comma-separated IDs. Do not include explanations or verifications in your output.  First, I will show the objectives that should be experienced.  # Objectives  ## Objectives: Symptoms  item,id  Fever,JlAK6lk  General malaise,JlAK6lo  Anorexia,JlAK6ls  Weight loss,JlAK6lw  Weight gain,JlAK6l0  Altered mental status,JlAK6l4  Syncope,JlAK6l8  Seizure,JlAK6mA  Vertigo and dizziness,JlAK6mE  Edema,JlAK6mI  Rash,JlAK6mM  Cough and sputum production,JlAK6mQ  Blood in sputum and hemoptysis,JlAK6mU  Dyspnea,JlAK6mY  Chest pain,JlAK6mc  Palpitations,JlAK6mg  Dysphagia,JlAK6mk  Abdominal pain,JlAK6mo  Nausea and vomiting,JlAK6ms  Hematemesis,JlAK6mw  Melena,JlAK6m0  Constipation,JlAK6m4  Diarrhea,JlAK6m8  Jaundice,JlAK6nA  Abdominal distention and abdominal mass,JlAK6nE  Lymphadenopathy,JlAK6nI  Abnormal urine output/urination,JlAK6nM  Hematuria,JlAK6nQ  Menstrual abnormality,JlAK6nU  Anxiety/depression,JlAK6nY  Cognitive dysfunction,JlAK6nc  Headache,JlAK6ng  Skeletal muscle paralysis/muscle weakness,JlAK6nk  Gait disturbance,JlAK6no  Sensory disturbance,JlAK6ns  Back pain,JlAK6nw  Arthralgia/joint swelling,JlAK6n0  ## Objectives: Major clinical and diagnostic imaging tests  item,id  Full blood count,JkxirwY  Blood biochemistry,Jli6WW4  Coagulation/fibrinolysis,Jli6WW8  Immunoserology tests,Jli6WXE  Urinalysis,Jli6WXI  Stool (fecal) examination,Jli6WXQ  Blood typing (ABO, RhD), blood compatibility test (cross-matching), atypical antibody screening,Jkxir08  Arterial blood gas analysis,JkxirxY  Pregnancy test,Jkxiryk  Microbiological tests (bacterial smear, culture, identification, antibiotic sensitivity test),JkxirxU  Cerebrospinal fluid,Jkxirxc  Pleural fluid analysis,Jli6TOw  Peritoneal fluid analysis,Jli6TO0  Histopathology and cytology (including intraoperative rapid diagnosis),Jkxirwk  Genetic testing and chromosome analysis,Jkxirwg  ECG,JkxirxQ  Lung function tests,Jli6Y7U  Endocrine and metabolic function tests,Jli6Y7Y  Electroencephalography,Jli6Y7c  Ultrasound,JkxirxA  X-ray,Jkxirw4  CT,Jli6aBM  MRI,Jli6aBU  Nuclear medicine examination,Jkxirxk  Endoscopy,Jkxirw8  ## Objectives: Basic clinical techniques  item id  Position change, transfer JlAKx_k  Skin antisepsis Jub5cSY  Application of topical medications Jub5cSc  Airway suction JlAKx_s  Nebulizer Jub5dh8  Venous blood sampling JlAKx_w  Peripheral venous catheterization JlAKx_0  Insertion and extraction of nasogastric tube JlAKyAA  Insertion and extraction of urinary catheter JlAKyAE  Intradermal injection JlAKyAI  Subcutaneous injection JlAKyAQ  Intramuscular injection JlAKyAU  Intravenous injection JlAKyAY  Urinalysis (including pregnancy test) Jub5eUA  Microbiological testing (including gram staining) JlAKyAc  Recording of a 12-lead ECG JlAKyAg  Rapid bedside ultrasound (including FAST) for clinical decision-making JlAKyAk  Rapid antigen/pathogen testing JlAKyAs  Blood glucose test JlAKyAw  Aseptic technique JlAKyA0  Surgical hand washing JlAKyA4  Gowning techniques in the operating room JlAKyA8  Basic sutures and suture removal JlAKyBA  Below, I present the clinical clerkship record of a medical student.  # clinical clerkship record  <clinical clerkship record here> |
| --- |
